# Becoming nose‐blind—Climate change impacts on chemical communication

**DOI:** 10.1111/gcb.16209

**Published:** 2022-05-16

**Authors:** Christina C. Roggatz, Mahasweta Saha, Solène Blanchard, Paula Schirrmacher, Patrick Fink, François Verheggen, Jörg D. Hardege

**Affiliations:** ^1^ Energy and Environment Institute University of Hull Hull UK; ^2^ Plymouth Marine Laboratory Plymouth UK; ^3^ Department of Chemical and Behavioural Ecology, Gembloux Agro‐Bio Tech Université de Liège Gembloux Belgium; ^4^ Department of Biological and Marine Sciences University of Hull Hull UK; ^5^ Department River Ecology Helmholtz Centre for Environmental Research GmbH – UFZ Magdeburg Germany; ^6^ Department Aquatic Ecosystem Analysis and Management Helmholtz Centre for Environmental Research GmbH – UFZ Magdeburg Germany

**Keywords:** behavior, chemoreception, climate change stressor, ecological interactions, infochemicals, ocean acidification, semiochemicals, sensory ecology

## Abstract

Chemical communication via infochemicals plays a pivotal role in ecological interactions, allowing organisms to sense their environment, locate predators, food, habitats, or mates. A growing number of studies suggest that climate change‐associated stressors can modify these chemically mediated interactions, causing info‐disruption that scales up to the ecosystem level. However, our understanding of the underlying mechanisms is scarce. Evidenced by a range of examples, we illustrate in this opinion piece that climate change affects different realms in similar patterns, from molecular to ecosystem‐wide levels. We assess the importance of different stressors for terrestrial, freshwater, and marine ecosystems and propose a systematic approach to address highlighted knowledge gaps and cross‐disciplinary research avenues.

## Graphical abstract

Chemical communication plays an essential role in ecosystems as it enables organisms to sense their environment, locate predators, food, habitats, or mates and interact with each other. We show that climate change can affect every single step of this fundamental way of communicating from the molecules to ecosystem‐wide interactions. Combined examples from terrestrial, freshwater and marine systems provide an overview across different realms and reveal universal patterns of impact, which help to identify the key aspects that we urgently need to understand to grow our ability to predict future effects and reliably inform ecosystem management efforts.

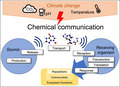

## 1 ALTERED CHEMICALLY MEDIATED INTERACTIONS—DOES CLIMATE CHANGE CHALLENGE THE “LANGUAGE OF LIFE”?

Chemically mediated interaction through so‐called infochemicals (Hay, 2009; Saha et al., 2019), often also referred to as semiochemicals, is arguably the oldest and most widespread form of communication (Wyatt, 2003). Infochemicals provide the basis for the vast majority of ecological processes across the tree of life in both terrestrial and aquatic ecosystems (Brönmark & Hansson, 2012; Wyatt, 2014), serving as cues or signals released into the surroundings or present on the surface of organisms (Wyatt, 2014). They cover a broad range of functions, mediating behaviors in trophic and non‐trophic interactions, such as predator–prey relationships (Ferrari et al., 2010), foraging and feeding‐deterrence (Kamio & Derby, 2017), habitat selection (Buxton et al., 2020), mate recognition and reproduction (Groot & Zizzari, 2019; Wyatt, 2014). Chemical communication shapes the structure and functioning of terrestrial (Hentley & Wade, 2016), freshwater (Burks & Lodge, 2002) and marine (Hay, 2009) ecosystems, maintaining their equilibrium (Sentis et al., 2015) and providing crucial ecosystem services that are of great importance to humans (Parachnowitsch & Manson, 2015).

Since the beginning of the industrial era, increasing atmospheric greenhouse gas concentrations give rise to climate change and affect a wide range of environmental parameters (IPCC, 2021). Increasing atmospheric carbon dioxide (CO_2_) concentrations affect terrestrial ecosystems and are partly absorbed by the world's waterbodies, where some of the CO_2_ reacts with water, forming carbonic acid that dissociates in equilibrium with the pH conditions depending on the dissolved carbonate buffer system (Wetzel, 2011). Hence, CO_2_ can have direct impacts on organisms in aquatic systems (hypercapnia) and indirect effects through a reduction of water pH (acidification) (IPCC, 2021), adding pH as a potential separate stressor. In addition, average air and water surface temperatures are rising, and extreme events like heatwaves are increasing in frequency, duration, and severity, which affects terrestrial and aquatic ecosystems alike (IPCC, 2021).

In recent years, evidence for severe impacts of future CO_2_ levels on a wide range of organisms' behaviors has grown (Clements & Hunt, 2015). However, results turn out to be often system‐specific, tricky to reproduce and dependent on the methodology and context of the behavioral assay and conditions (Clark et al., 2020a). This recently sparked a push for more robust methods and comparability through transparent observation and objective parameter determination (Clark et al., 2020b; Munday et al., 2020), but also highlights a lack of understanding of the underlying mechanisms.

**FIGURE 1:**
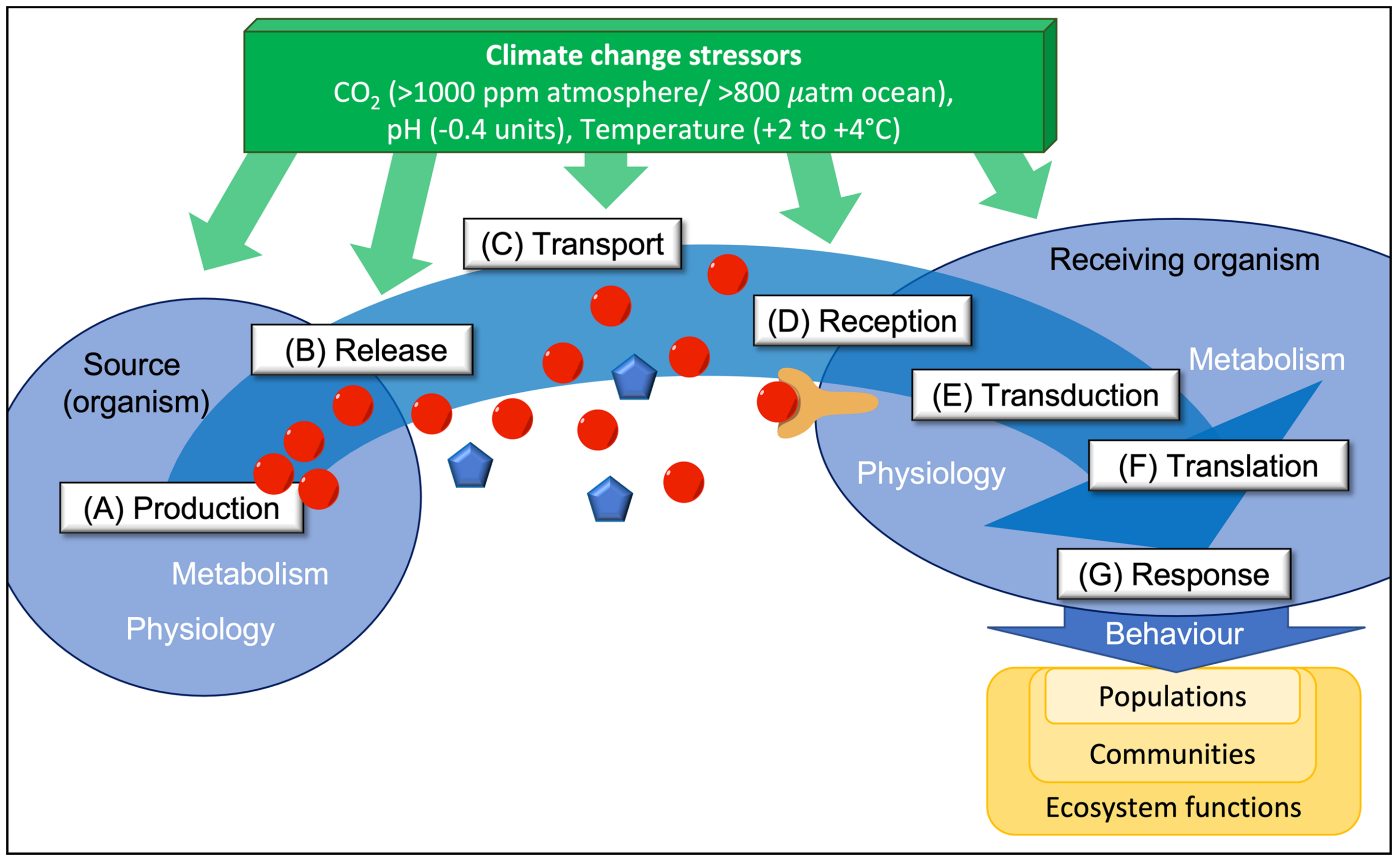
Schematic framework of the steps within the chemical communication process that are potentially sensitive to climate change stressors. Successful chemical communication via infochemicals (red circles, often also referred to as semiochemicals) requires a multitude of different subsequent steps. It starts with signal production (A) and the release of the infochemical (B), followed by the transport of the signal through water or air (C) and reception by another organism (D). Within the receiver, the signal is transduced (E) and translated (F) before it leads to a physiological or behavioral response (G). All these steps can be altered directly or indirectly through CO_2_ concentration, pH, and/or temperature, which will ultimately jeopardize the communication process. Direct effects are particularly relevant during steps (C) and (D), for example, changes to the infochemicals (blue pentagons instead of red circles). Indirect effects are mainly caused by stressors altering the physiology and metabolism of an organism, which in turn affects steps (A), (B), and (E) to (G). Resulting changes in behavior can affect populations and communities by influencing intra‐ and inter specific interactions, and have cascading implications for the stable functioning of ecosystems. Impacting stressors (green box) are specified based on latest IPCC predictions (IPCC, 2021)

It is striking that many of the behaviors affected by CO_2_ fundamentally rely on the successful reception and interpretation of chemical information. This chemical communication process involves several steps at different scales from molecular level to whole organisms (Figure 1). After the infochemicals are produced (A) and released (B) by an organism or other source, they are transported through (or in contact with) water or air (C), before being received by another organism (D). After transducing (E) and translating (F) the neuronal signal, the receiving organism responds through a measurable physiological or behavioral change (G). In this opinion piece, we present multiple lines of evidence from different ecosystems that each step in this communication process may be altered (positively or negatively) by climate change‐associated stressors.

## 2 EVIDENCE FROM DIFFERENT REALMS: CLIMATE CHANGE IMPACTS ON THE CHEMICAL COMMUNICATION PROCESS

### 2.1 The terrestrial realm

**FIGURE 2:**
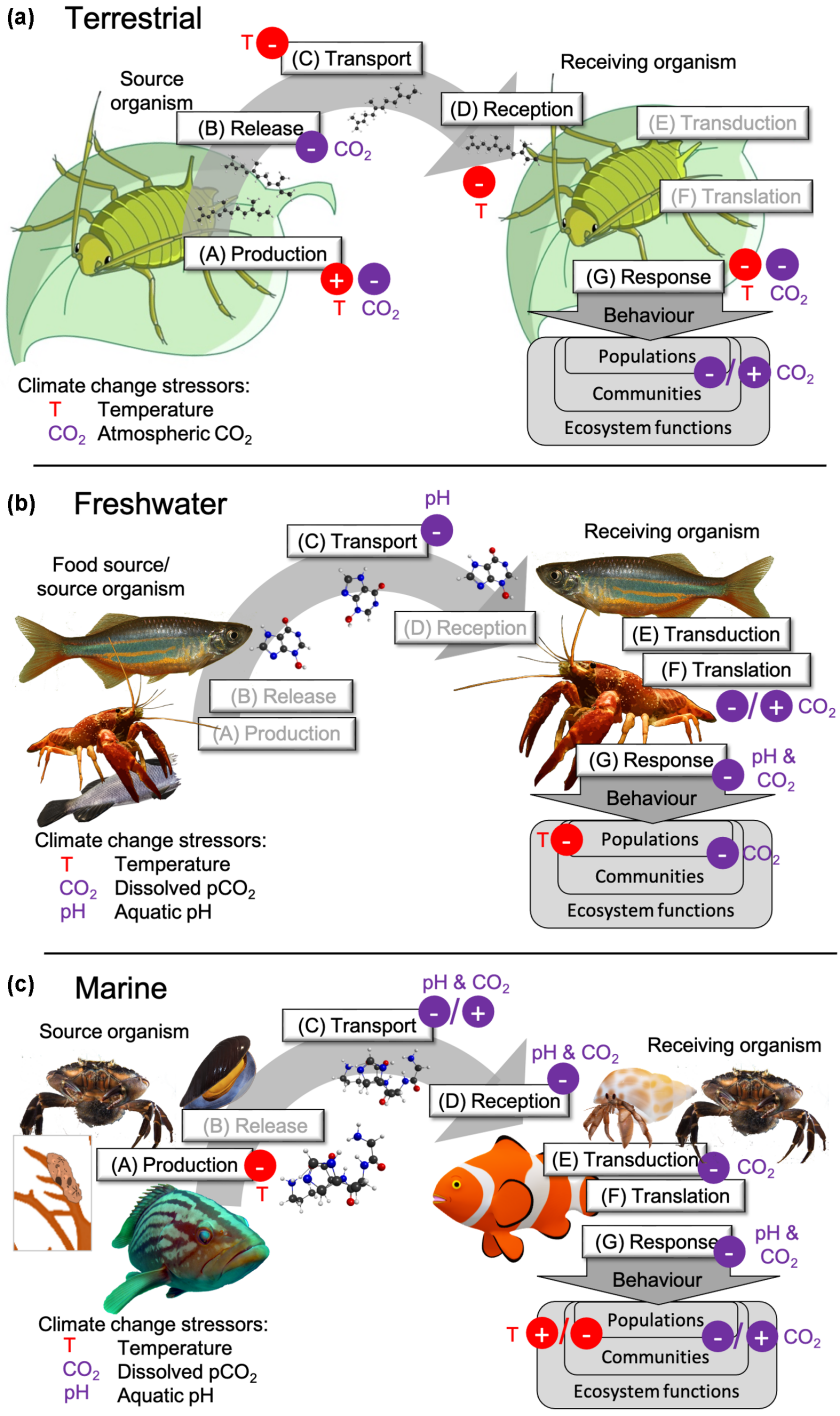
Impacts of climate change stressors on individual steps of the chemical communication process based on examples for (a) terrestrial, (b) freshwater, and (c) marine systems. Steps follow the scheme in Figure 1, but infochemical sources and receivers vary depending on system. Relevant stressors are indicated and defined in the bottom left corner of each panel. Icons in the respective stressor color indicate positive (+) or negative (−) effects on this step, which are described in detail in Section 2. Communication steps for which relevant studies are missing are marked in gray

Insects are heavily dependent on their olfactory system to interpret their environment and rely on infochemicals for intra‐ and interspecific communication. Because insects are poikilotherms and ectotherms, thermal stress can impact internal enzymatic activities, with cascading effects on biosynthesis and chemical composition of pheromones [(A) in Figure 2a] (Groot & Zizzari, 2019). For instance, ladybeetle larvae produce twice as much infochemical under 25°C than under 15°C (Sentis et al., 2015). Once emitted, sudden shifts in environmental temperature can accelerate pheromone decay (van Oudenhove et al., 2011) (C) and reduce pheromone detectability by insects as a consequence of disturbed activation of the olfactory receptors (Groot & Zizzari, 2019) (D). These disruptions in chemical communication can cause a reduction in individuals' ability to locate each other at a distance and identify potential mates.

Elevated CO_2_ levels further impact the pheromone‐mediated predator response in aphids. Pea aphids reared under elevated CO_2_ concentration (800 ppm) were found to produce ≈12% less of their alarm pheromone than under control CO_2_ concentration (450 ppm) (Boullis et al., 2017), resulting in the release of ≈35% less pheromone in case of attack by a predator (A and B). This could result from modifications of the host plant sap (Chen et al., 2019) where pheromone precursors may be collected by aphids during their feeding and development. The aphids' ability to perceive the pheromone was not affected by CO_2_ (D) (Boullis et al., 2017). However, the escape behavior exhibited by aphids exposed to the alarm pheromone is inhibited when they are grown under enriched CO_2_ environment or under higher temperatures, as demonstrated for different aphid species (G) (Boullis et al., 2017; Hentley et al., 2014; Mondor et al., 2004; Sun et al., 2010). Contradictory results were obtained among different aphid strains, confirming that atmospheric conditions may alter genotypic and phenotypic frequencies of herbivore populations (Mondor et al., 2005), with contrasting consequences on intraspecific chemical communication (Boullis et al., 2017).

Modified atmospheric gas composition was also found to impact the infochemical‐dependent interactions between aphids and other insects (Boullis, Francis, et al., 2018). While the kairomones released by aphid honeydew are qualitatively and quantitatively modified under elevated CO_2_ concentration (A), no difference was observed in the searching and oviposition behaviors of the predatory hoverfly (*Episyrphus balteatus*) (Boullis, Blanchard, et al., 2018; Boullis, Francis, et al., 2018) (G). However, the number of eggs laid on an aphid colony by this predatory species is lowered under elevated CO_2_ concentrations (Boullis, Blanchard, et al., 2018; Boullis, Francis, et al., 2018) (G), probably as a result of the reduction in the emission of alarm pheromone (Verheggen et al., 2008). Climate change will also beneficially impact the semiochemistry of aphid‐ant mutualism (Blanchard et al., 2019; Verheggen et al., 2012). Under elevated temperatures, ants walked more actively through an aphid colony, providing increased care and defence to their partners while receiving more honeydew in exchange (Blanchard et al., 2021). These studies illustrate the complex effects of increased temperature and CO_2_ concentrations on multitrophic interactions and demonstrate the idiosyncratic characteristics of natural enemy responses (Boullis et al., 2015, 2016).

### 2.2 The freshwater realm

Despite the growing awareness of the consequences of rising atmospheric CO_2_ concentrations on freshwater ecosystems, only very few studies have investigated potential effects on chemical communication processes beyond some species of fish and crustaceans (Figure 2b) (Hasler et al., 2018; Leduc et al., 2013).

Research in the 1980s and 90s on consequences of freshwater acidification through acid rain found crayfish to have impaired ability to locate food in pH conditions lower than in their natural habitat (Allison et al., 1992; Tierney & Atema, 1986) (G). Similar findings were made for foraging‐associated behaviors in newts (Griffiths, 1993) and fish (Tembo, 2009) (G). Recently, increases in dissolved CO_2_ were shown to reduce the formation of a kairomone‐induced morphological antipredator defence in the freshwater zooplankton *Daphnia* sp. (Weiss, Pötter, et al., 2018). The defence‐inducing signals are amino conjugates of fatty acids (Weiss, Albada, et al., 2018), but it remains unclear how elevated CO_2_ levels impact this cue or its perception by *Daphnia*. Temperature also influences kairomone‐induced defences of *Daphnia*, and higher temperatures in combination with predator cues increased mortality rates (Hanazato, 1991).

Fish use chemical cues both for intra‐ and interspecific communication, for example to avoid predators (Ferrari et al., 2010). Acidification, both by the addition of sulfuric acid (Brown et al., 2012) and atmospheric CO_2_ (Ou et al., 2015), reduces the anti‐predator behavior of juvenile fish and/or decreases their anxiety levels towards potential predators (G). One particularly well studied example for chemical communication in fish are alarm cues (“schreckstoff”) found across many fish species (Wisenden, 2000). The most commonly discussed schreckstoff, hypoxanthine‐3‐N‐oxide, undergoes irreversible structural changes at pH levels below 6.0 (C), which prevents its detection by fish (Brown et al., 2002). However, the associated reduction in behavioral response (G) appears to be specific to certain fish species (Tix et al., 2017).

Molecular changes to the infochemicals (C) were previously hypothesized as the main route of how CO_2_ affects chemosensory responses of freshwater organisms (Leduc et al., 2013). However, there is also evidence for interference with neurotransmitter functioning through altered sensitivity of GABA receptors (E and F) in freshwater snails and crustaceans (Abboud et al., 2019), a mechanism initially only thought to affect marine systems.

### 2.3 The marine realm

Over the past decade, marine studies increasingly focussed on climate change‐related disruption of behaviors and interactions mediated through olfactory systems and infochemicals (Clements & Hunt, 2015; Draper & Weissburg, 2019), but only few investigated the actual underlying processes (Figure 2c).

Marine macrophytes depend on chemical communication for defence against colonizers (Saha et al., 2018), predators (Toth & Pavia, 2000) and disease‐causing pathogens (Saha & Weinberger, 2019). In red seaweed *Delisea pulchra* under high temperature conditions, bleached thalli had lower levels of antibacterial defence than healthy thalli due to a decreased production of halogenated furanones (Campbell et al., 2011) (A), which function as quorum sensing inhibitor compounds (Manefield et al., 2002). Similarly, in *Laurencia dendroidea* (Sudatti et al., 2011), high temperature and salinity (30°C, 40 psu) can lead to decreased elatol production (A), a sesquiterpene that can inhibit herbivory (Pereira et al., 2003) and settlement of biofoulers (Da Gama et al., 2003). However, extreme events like heatwaves do not jeopardize the chemical defence of the brown seaweed *Fucus vesiculosus* against bacteria and the seagrass *Zostera marina* against the pathogen *Labyrinthula zosterae* (Saha et al., 2020), but upregulate the defence capacity of *Zostera* against surface bacterial colonization (Guan et al., 2020).

A range of marine crustaceans and molluscs use peptides with signalling function to coordinate brood‐care, homing, and settlement (Rittschof & Cohen, 2004). These peptides are changed reversibly by a difference of only 0.4 pH units (Roggatz et al., 2016) (C). Reduced pH increases the proportion of protonated signal molecules, which significantly differ in charge distribution and three‐dimensional conformation from non‐protonated molecules (Roggatz et al., 2016). This translates directly into a reversibly altered behavioral response by female *Carcinus maenas* crabs, exhibiting reduced egg‐ventilation behavior and an increased response threshold in lower pH conditions (G) (Roggatz et al., 2016). Tetrodotoxin and saxitoxin, two of the most potent biotoxins, are used in predator deterrence for species ranging from pufferfish to dinoflagellates and become protonated (C), hence more potent, at a global scale in predicted future oceanic conditions (Roggatz, Fletcher, et al., 2019).

Electrophysiological and transcriptomic measurements show that elevated CO_2_ levels impair the olfactory system of sea bass (Porteus et al., 2018) and sea bream (Velez et al., 2019) (D). The altered chemoreception can be attributed to changes in the infochemical (C), the olfactory receptor or the olfactory epithelium (Schirrmacher et al., 2021; Velez et al., 2019) (D). Additionally, ocean acidification has been shown to interfere with neurotransmitter functioning as the internal compensation for elevated CO_2_ conditions can lead to altered brain ion gradients (Nilsson et al., 2012) (E and F). Treatment with GABA_A_ antagonists reversed olfactory disruption in larval coral reef fish (Nilsson et al., 2012), but not in hermit crabs (de la Haye et al., 2012). Asian shore crab larvae decreased kairomone identification on gabazine treatment (Charpentier & Cohen, 2016), indicating that species‐ and system‐specific mechanisms are involved. Despite inhabiting coastal environments with very changeable conditions (de la Haye et al., 2012), a pH reduction of only 0.6 units significantly reduces hermit crabs' (*Pagurus bernhardus*) effectiveness and speed of their foraging behavior in response to filtered mussel extract (Roggatz, Kenningham, et al., 2019) and at pH 6.8 also towards fish extract (de la Haye et al., 2012) (G).

Chemical communication at the community level can be extremely complex and is therefore rarely studied. Valuable insights are provided by recent studies on the community of seagrass meadows (*Posidonia oceanica*) and their associated epiphytes (Maibam et al., 2014; Mutalipassi et al., 2020; Zupo et al., 2015, 2016). Here, grazing on seagrass and/or its epiphytes causes the release of volatile organic compounds (VOCs) (Maibam et al., 2014). Recent evidence shows that ocean acidification alters the bouquet of released cues from the Posidonia‐epiphyte system compared with normal seawater pH (Mutalipassi et al., 2022). These infochemicals are used by invertebrates associated with the seagrass‐community and induce chemotactic reactions, which differ between current and high CO_2_ conditions (Zupo et al., 2015, 2016). Behavioral differences match differences in the abundance of invertebrates in natural vs. acidified field conditions (Maibam et al., 2014). Seawater acidification therefore was shown to influence the emitted bouquet (A) and chemoreception of plant‐produced infochemicals (G) and the structure of the epifaunal communities (Mutalipassi et al., 2022; Zupo et al., 2015, 2016). Diatoms release volatile aldehydes that inhibit the population growth of marine zooplankton, the most abundant animal group on our planet (Ianora et al., 2004). Copepod zooplankton use VOCs to discern between beneficial algae and aldehyde‐releasing diatoms (Maibam et al., 2015). However, under ocean acidification conditions, the copepods' preference for the harmless algae is reduced, whereas the attractivity of the VOCs from the aldehyde‐producing (more harmful) diatoms increases (Maibam et al., 2015). Such intricate changes in adaptive decision‐making may be some of the easily overlooked effects of ocean acidification that can have ecosystem‐wide consequences.

## 3 THE EMERGING BIGGER PICTURE—SYSTEM‐SPECIFIC STRESSORS, A CASCADE OF AFFECTED COMMUNICATION STEPS AND THEIR IMPLICATIONS

Combining the realm‐specific studies confirms our suggestion that climate change–associated stressors have the potential to disrupt chemical communication at every step of the signalling cascade. Similar patterns can be identified across all realms (Figure 2). Only few studies show potentially positive effects of climate change stressors on the chemical communication process (Roggatz, Fletcher, et al., 2019; Sentis et al., 2015) or hint at resilience (Boullis et al., 2017; Saha et al., 2020), and most behaviors associated with infochemicals are clearly impaired or altered (Clements & Hunt, 2015; Mutalipassi et al., 2022; Zupo et al., 2015). These effects happen primarily at the individual level, but also cascade up to population, community and ecosystem levels through impacts on brood‐care and settlement (Roggatz et al., 2016), anti‐predator behavior (Brown et al., 2012; Chivers et al., 2014; Ou et al., 2015), foraging (Roggatz, Kenningham, et al., 2019; Tierney & Atema, 1986; Velez et al., 2019), host–parasite interactions (Furlong & Zalucki, 2017; Senior et al., 2020) and chemically mediated community structure (Zupo et al., 2015, 2016).

As Figure 2 shows, the impact of the different stressors varies with each system. Stressors have direct effects on synthesis, transport, and reception of the infochemical or indirectly impact processes in the source and the receiving organisms by affecting their physiology or metabolism. Increased temperature and atmospheric CO_2_ levels are the main stressors in terrestrial environments, acting separately or synergistically with mainly negative consequences for the chemical communication process except for some indirect positive effects on cue production. In freshwater systems, pH and dissolved inorganic carbon have been identified as main stressors with negative direct and indirect effects. The aquatic carbonate equilibrium can be affected by photosynthesis and respiration (diurnal pH fluctuations) (Baumann & Smith, 2018), high NO_x_ and SO_2_ emissions (acid rain) (Schindler, 1988) and local catchment geology (Wetzel, 2011) of freshwater systems, and tides in marine systems (Wolfe et al., 2020). In the marine realm all three stressors, pH, dissolved CO_2,_ and temperature, were found to impact chemical communication mostly negatively, although there are some positive direct and indirect effects. Combined stressor experiments on chemically mediated interactions are currently very scarce and only provide a glimpse at a complex interplay affecting observable behaviors (see aphid alarm behavior (Boullis et al., 2017) and *Daphnia* defences (Hanazato, 1991)).

The predominantly negative effects of climate change on chemical communication suggest the potential for cascading negative impacts in terrestrial and aquatic ecosystems with a range of far‐reaching implications. Negative effects on key interactions, such as mutualistic plant–pollinator interactions (Kiers et al., 2010), can threaten essential ecosystem services like pollination (Vanderplanck et al., 2021). Consequences of altered mutualistic interactions are further unlikely to remain limited to directly involved organisms but will likely expand to other organisms within the community (Zupo et al., 2015, 2016). This will accelerate the effects of global change on biodiversity loss and ecosystem disruption (Kiers et al., 2010). In addition, further economic issues may arise through the proliferation of pest and invasive species, most of which are expected to be positively impacted by climate change (Jactel et al., 2005). Impacts for the fishing industry due to the disruption of key behaviors, such as larval settlement or foraging, in economically important fish, mollusc and crustacean species are also foreseeable (Porteus et al., 2018; Roggatz et al., 2016). With crucial ecosystem services and processes at stake, we need to start addressing and understanding the underlying mechanisms that alter behaviors and interactions in a more cross‐disciplinary way.

## 4 CALLING FOR CROSS‐DISCIPLINARY COLLABORATION WITH A SYSTEMATIC APPROACH TO ENHANCE OUR PREDICTIVE CAPACITY

Interactions and behaviors altered under climate change scenarios can be directly or indirectly linked to changes of the chemical communication process in a universal way that applies across the very different realms, as shown here (Figure 2). Our systematic approach of impact assessment enabled us to identify the current knowledge gaps. We propose our framework as a starting point to allow for more holistic, cross‐realm informed and generally applicable investigations of climate change impacts on chemically mediated processes in ecosystems. This will require multidisciplinary collaborations to enhance our mechanistic understanding and cover a range of essential aspects with regard to the experimental setups used, timescales investigated and environmental contexts of the studied system in future investigations.

Although for terrestrial ecosystems many infochemicals are well characterized, marine and particularly freshwater systems suffer from a significant lack of fully elucidated chemical structures that can be directly linked to measurable behavioral or physiological responses. The widespread use of mainly unidentified molecules and complex mixtures of unknown concentrations in experiments, especially in aquatic studies, severely limits the insights gained at the mechanistic level and prevents systematic investigations at the ecosystem scale. Increased close collaboration between natural product chemists and ecologists to identify infochemicals and their ecological roles is essential to establish better cross‐applicable systems. In addition, for those few structurally elucidated systems, we lack crucial chemical and physical understanding of how stressors alter the chemical structure of either the infochemicals or their respective receptors, or in fact where the receptors are located and how they function. Here, close collaborations between biochemists, molecular biologists, sensory physiologists, and computational modellers are urgently required to cross realm and discipline boundaries. Findings for insects with known receptor systems (as summarized for example by Leal, 2013) might be transferable to aquatic arthropods for which molecular information on receptors is exceptionally scarce (Kozma et al., 2018).

Comparative assessment of the importance of these direct impacts versus indirect influences through physiological and metabolic stress affecting organism fitness or ability to process information will be essential. This would help to establish the levels of individual variability in light of different stressors. Combining evidence from laboratory and field studies can then address how relevant individual effects and variability are in the context of complex natural systems, which currently presents a key bottleneck. Mesocosm approaches are useful to bridge the scaling gap between mechanistic laboratory assays and complex natural systems (e.g., Engel et al., 2005; Fink et al., 2020). Field sites, such as natural CO_2_ vents and seeps or upwelling areas, provide further complementary insights into short and long‐term effects (Foo et al., 2018). The species and system specificity of the case studies collated above severely limits our mechanistic understanding across all steps and levels of the communication process. Applying our proposed systematic approach to a wider range of species and systems would allow to investigate climate change impacts on the entire process from the communication molecule to the ecosystem function.

Current studies are further based on a whole range of different setups, a multitude of incomparably measured parameters and a variety of different conditions (Clark et al., 2020a). Reproducibility and comparability of studies could be improved through blind analysis and publication of different quantifiable parameters, for example by recording and analysing videos alongside qualitative behavioral observations (outcome of a choice experiment, type of feeding behavior). To reduce the likelihood of a ‘decline effect’, pre‐registration of studies could provide a useful avenue (Clements et al., 2022). Consistent definitions of stressor ranges in agreement with IPCC scenarios (IPCC, 2021) would enable more meaningful cross‐study conclusions. In aquatic systems, investigations of temperature effects on chemically mediated processes are particularly underrepresented. In addition, local, short, climatic extreme events, such as heatwaves, may have even greater impacts than long‐term average changes (Ulseth et al., 2018), warranting particular attention and demonstrating the need for the investigation of different timescales and the respectively relevant stressor ranges in combination. Currently, the local environmental context of a study organism is often neglected in favor of average current and future global study conditions. Studying impacts of local stressor parameters instead and comparing organisms from different origins or various local environments would aid the systematic understanding with regard to phenotypic plasticity, acclimation, and adaptation potential, extending on the previously proposed aspects by Sunday et al. (2014). Adaptation or at least acclimatization of some steps within the signalling cascade may be possible, but physiological and behavioral adaptation seems to be favored over olfactory plasticity based on investigations in *Platynereis* sp. polychaetes (Calosi et al., 2013; Lucey et al., 2015; Wäge et al., 2017). The physiological and evolutionary potential for adaptation might also be realm‐specific based on how commonly fluctuations in current abiotic conditions occur. Using local source and receiver organisms subjected to the same stressor(s) and comparing constant, naturally relevant fluctuating, and brief extreme environmental conditions would significantly advance our knowledge base. This is particularly important for organisms inhabiting highly variable or extreme ecosystems.

Combined, all these insights will allow us to answer whether, how and in which way altered or disrupted aspects of the chemical communication process will impact behaviors and interactions and therefore the stability and resilience of ecosystems and the services they provide (Box 1 for a summary of selected outstanding questions). Consistency in concepts and approaches across biomes and types of interactions will increase the predictive power and generalizability of these approaches.

BOX 1 **Outstanding questions from molecular to ecosystem scale**

- Does the class of chemical compounds mediating a specific interaction play a crucial part in the sensitivity of this interaction to climate change stressors?
- How vulnerable are olfactory receptors to climate change stressors?
- How much plasticity and adaptation potential is there for each step of the signalling cascade in the chemical communication process?
- Can organisms learn or evolve a new ‘chemical language of life’ by switching to a different (set of) chemical(s) for the same information/ communication purpose? Could they even switch to a different sensory mode (e.g. visual or mechanical cues)?
- Are organisms that live in environments with larger fluctuations of environmental conditions (e.g. in freshwater or intertidal systems) less susceptible to climate change than their counterparts in more stable ecosystems, despite using similar infochemicals?
- How is altered chemical communication reflected in complex multi‐trophic and community systems?
- What is the impact of disrupted chemical communication on ecosystem services? Are there direct, quantifiable links?

There also is a significant lack of dedicated studies for more complex multitrophic and community systems and the impact of disrupted chemical communication on ecosystem services, limiting the application of predictive systematic modelling. Localizing studies and modelling can aid in identifying geographic stressor hotspots (Roggatz, Fletcher, et al., 2019) that influence keystone species and communities. This can fundamentally guide ecosystem management approaches and industry, for example to pre‐empt vulnerable locations and times that could be forecasted based on predicted local changes in acidity (Brady et al., 2020). This ultimately facilitates climate change mitigation and ecosystem management by helping to inform decision‐makers.

## 5 CONCLUSION

As it becomes evident that climate change can affect all steps of the chemical communication process, we call for a systematic and comprehensive approach combining theoretical and experimental aspects. Understanding the underlying mechanisms and how they translate to effects on ecosystem stability and resilience or affect services will require joint and multidisciplinary efforts across realm boundaries. Studies combining our suggested framework with a future focus on the indicated aspects and outstanding questions (Box 1) will significantly enhance the predictive power of chemical ecological research and help to inform management strategies for terrestrial, freshwater, and marine ecosystems alike.

## CONFLICT OF INTEREST

No competing interests.

## AUTHOR CONTRIBUTIONS

**Christina C. Roggatz:** Conceptualization (lead); writing – original draft preparation (equal); writing – review and editing (lead). **Mahasweta Saha:** Conceptualization (support); writing – original draft preparation (equal), writing – review and editing (support); **Solène Blanchard:** Conceptualization (support); writing the draft (equal) and review and editing (support). **Paula Schirrmacher:** Conceptualization (support); writing the draft (equal) and review and editing (support). **Patrick Fink:** Conceptualization (support); writing the draft (equal) and review and editing (support); **François Verheggen:** Conceptualization (support); writing the draft (equal) and review and editing (support). **Jörg D. Hardege:** Conceptualization (support); writing the draft (equal) and review and editing (support).

## Data Availability

Data sharing not applicable to this article as no datasets were generated or analysed during the current study.
